# Polydeoxyribonucleotide Promotes Longitudinal Bone Growth Through the Hepatic JAK2-STAT5/IGF-1 Axis Involving A2A Receptor Signaling

**DOI:** 10.3390/molecules31193558

**Published:** 2026-10-07

**Authors:** Kyung-Min Jeong, Yu-Jin Choi, Seong-Been Yang, Minson Kweon, So-Young Rah, Hyun-Ja Jeong

**Affiliations:** 1 Department of Bio-Convergence System, Hoseo University, 20 Hoseo-ro, 79 Beon-gil, Baebang-eup, Asan 31499, Chungcheongnam-do, Republic of Korea; juyk10@naver.com (K.-M.J.); dbwls9638@naver.com (Y.-J.C.); 2 Department of Food and Biotechnology, Graduate School, Hoseo University, Baebang-eup, Asan 31499, Chungcheongnam-do, Republic of Korea; sbin1597@naver.com; 3 COSMAX NBT INC., Seongnam-si 13486, Gyeonggi-do, Republic of Korea; minson@cosmaxnbt.com; 4 Department of Biochemistry and Molecular Biology, Jeonbuk National University Medical School, 20 Geonji-ro, Deokjin-gu, Jeonju-si 54907, Jeollabuk-do, Republic of Korea

**Keywords:** bone growth, IGF-1, PDRN, A2AR, JAK2-STAT5 pathway, bone microarchitecture

## Abstract

Longitudinal bone growth is primarily regulated by the growth hormone (GH)-insulin-like growth factor-1 (IGF-1) axis. However, non-hormonal and orally administered strategies for promoting longitudinal growth remain limited. Here, we investigated the effects and mechanisms of polydeoxyribonucleotide (PDRN) on longitudinal bone growth in vitro and in vivo. In HepG2 cells, PDRN significantly increased IGF-1 mRNA expression and JAK2 and STAT5 phosphorylation without affecting cell viability. Pretreatment with ZM241385, a selective adenosine A2A receptor (A2AR) antagonist, significantly attenuated these responses, supporting the involvement of A2AR signaling. In mice, oral PDRN for 5 weeks significantly increased body length gain, growth plate thickness, and hepatic JAK2/STAT5 activation, elevating serum IGF-1 levels without altering GH levels or systemic toxicity markers. Quantitative micro-computed tomography analysis demonstrated improved trabecular and cortical bone microarchitecture with reduced porosity. Furthermore, PDRN modulated bone remodeling-related responses by suppressing lipopolysaccharide-induced osteoclastogenic marker expression in RAW 264.7 cells and enhancing osteogenic marker expression while downregulating RANKL in MC3T3-E1 cells. Overall, oral PDRN promotes longitudinal bone growth and improves microarchitecture via increased hepatic IGF-1 signaling and altered bone remodeling-related gene expression, supporting its potential as an oral candidate for skeletal growth.

## 1. Introduction

During childhood, linear skeletal development serves as a primary metric for overall physical growth, regulated by an intricate combination of endocrine, dietary, ecological, and hereditary determinants [1,2]. Disruptions in this developmental progression can lead to ramifications extending past somatic limitations, frequently resulting in psychological stress and compromised life quality [3,4].

Skeletal elongation predominantly takes place within the epiphyseal growth plate, a dynamic cartilaginous region where chondrocytes execute a tightly orchestrated sequence of proliferation, hypertrophic maturation, and eventual endochondral ossification [5,6]. This biological process relies on sophisticated cross-talk between systemic circulating factors and localized paracrine signals [7,8]. Key among these regulators are growth hormone (GH) and insulin-like growth factor-1 (IGF-1), which together orchestrate post-natal longitudinal growth. Upon binding to its specific receptor (GHR), GH triggers downstream signaling via the Janus kinase 2 (JAK2) and signal transducer and activator of transcription 5 (STAT5) cascade, thereby upregulating systemic IGF-1 synthesis in the liver [9,10]. Circulating and locally produced IGF-1 subsequently drives longitudinal skeletal accretion by accelerating chondrocyte expansion and terminal hypertrophy within the growth plate matrix [11,12,13].

Polydeoxyribonucleotide (PDRN) consists of a DNA-derived linear purine/pyrimidine polymer (molecular weight spanning 50–1500 kDa), typically extracted and purified from salmonid germ cells, such as *Oncorhynchus mykiss* or *Oncorhynchus keta* [14]. PDRN exhibits marked therapeutic attributes, including angiogenesis promotion, tissue remodeling acceleration, and anti-inflammatory activity, primarily mediated through adenosine A_2A_ receptor (A2AR) activation alongside salvage pathway purine recycling [15,16]. Given these biological functions, PDRN has been widely utilized in dermal restoration and soft tissue healing models [17]. Furthermore, accumulating evidence indicates that PDRN positively influences osteoblast lineage commitment and various osseous tissue responses in vitro [18,19,20]. Nevertheless, its potential efficacy in stimulating systemic longitudinal bone elongation in vivo has not yet been established.

Accordingly, the present study sought to determine whether systemic PDRN administration enhances longitudinal skeletal growth in a juvenile rodent model (growing ICR mice) and to elucidate the cellular pathways involved using HepG2, RAW 264.7, and MC3T3-E1 cell culture systems.

## 2. Results

### 2.1. PDRN Stimulates IGF-1 Expression via A2AR-Mediated JAK2-STAT5 Signaling in HepG2 Cells

**Figure 1:**
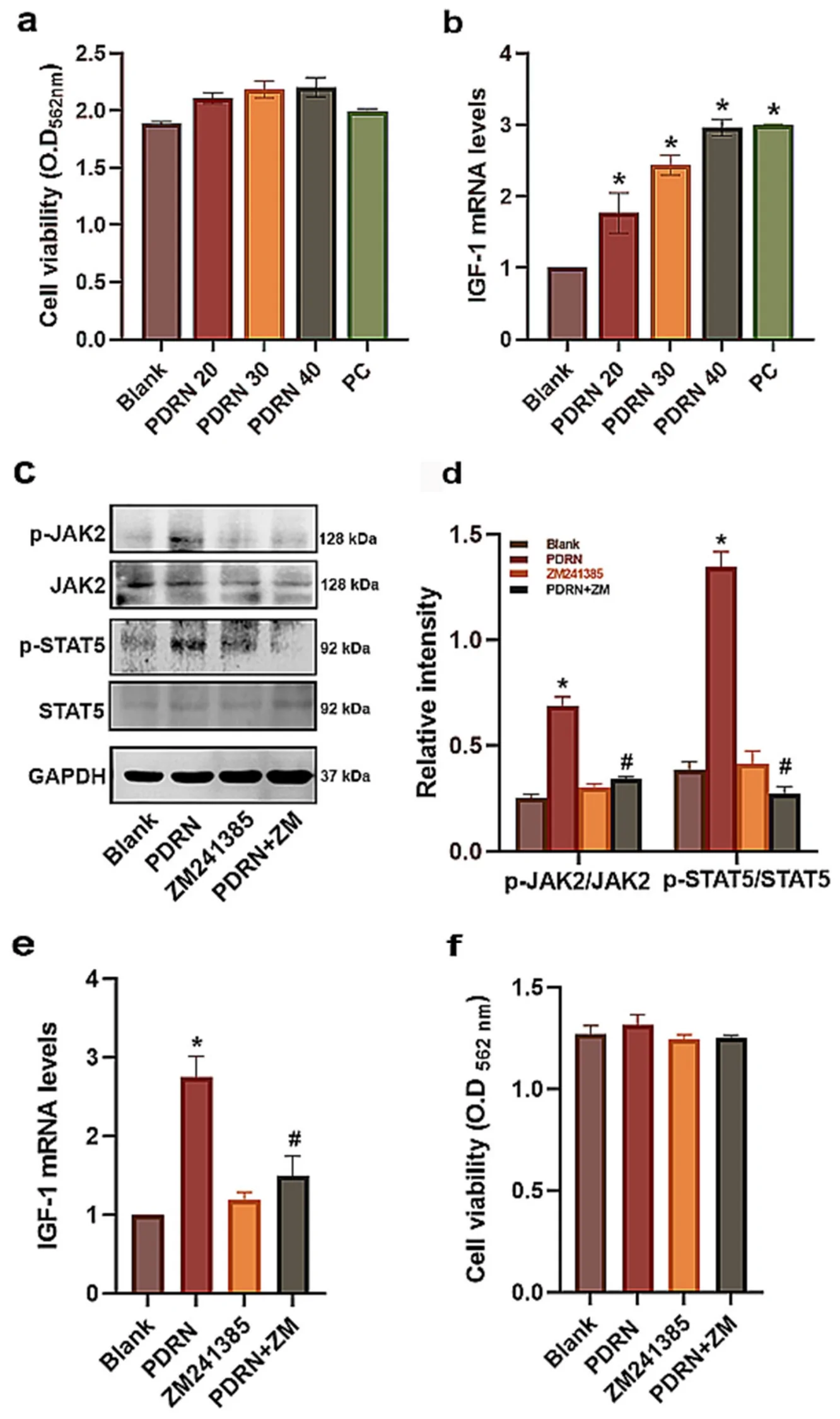
PDRN stimulates IGF-1 expression via the A_2A_ receptor-mediated JAK2-STAT5 pathway in HepG2 cells. (**a**) Cell viability assessed by MTT assay after 48 h treatment. (**b**) Relative IGF-1 mRNA expression determined by qRT-PCR after 24 h treatment. HepG2 cells were pretreated with ZM241385 (1 μM) for 30 min before stimulation with PDRN (40 μg/mL) for 24 h. (**c**) The levels of p-JAK2, JAK2, p-STAT5, and STAT5 were determined by Western blotting, with representative images shown. (**d**) Protein expression levels were quantified by densitometry and normalized to JAK2 and STAT5 (p-JAK2/JAK2 and p-STAT5/STAT5). (**e**) IGF-1 mRNA expression was assessed by qRT-PCR. (**f**) Cell viability assessed by the MTT assay. Data are presented as mean ± SEM (*n* = 3). * *p* < 0.05 vs. Blank; ^#^ *p* < 0.05 vs. PDRN 40. Blank, vehicle control; PC, positive control (100 μg/mL); PDRN + ZM, PDRN (40 μg/mL) + ZM241385 (1 μM).

To evaluate the potential cytotoxicity of PDRN, HepG2 cells were treated with various concentrations of PDRN or a positive control (PC). A 3-(4,5-dimethylthiazol-2-yl)-2,5-diphenyltetrazolium bromide (MTT) assay revealed no significant effects on cell viability (Figure 1a). Given the pivotal role of IGF-1 in linear bone growth [11], we examined the effect of PDRN on IGF-1 mRNA expression. PDRN significantly increased IGF-1 mRNA expression in a dose-dependent manner (Figure 1b; *p* < 0.05).

To further elucidate the upstream receptor mechanism underlying PDRN-induced signaling, we investigated the role of A2AR using its selective antagonist, ZM241385. PDRN markedly increased the phosphorylation levels of JAK2 and STAT5 (p-JAK2 and p-STAT5) without altering their total protein levels (Figure 1c,d). However, pretreatment with ZM241385 significantly suppressed the PDRN-induced upregulation of p-JAK2 and p-STAT5 (Figure 1c,d; *p* < 0.05). Consistently, the PDRN-mediated increase in IGF-1 mRNA expression was significantly attenuated by ZM241385 without inducing cytotoxicity (Figure 1e,f; *p* < 0.05). ZM241385 alone exerted no noticeable effect on baseline IGF-1 mRNA levels (Figure 1e).

### 2.2. PDRN Increases Hepatic IGF-1 Expression and Activates JAK2-STAT5 Signaling In Vivo

**Figure 2:**
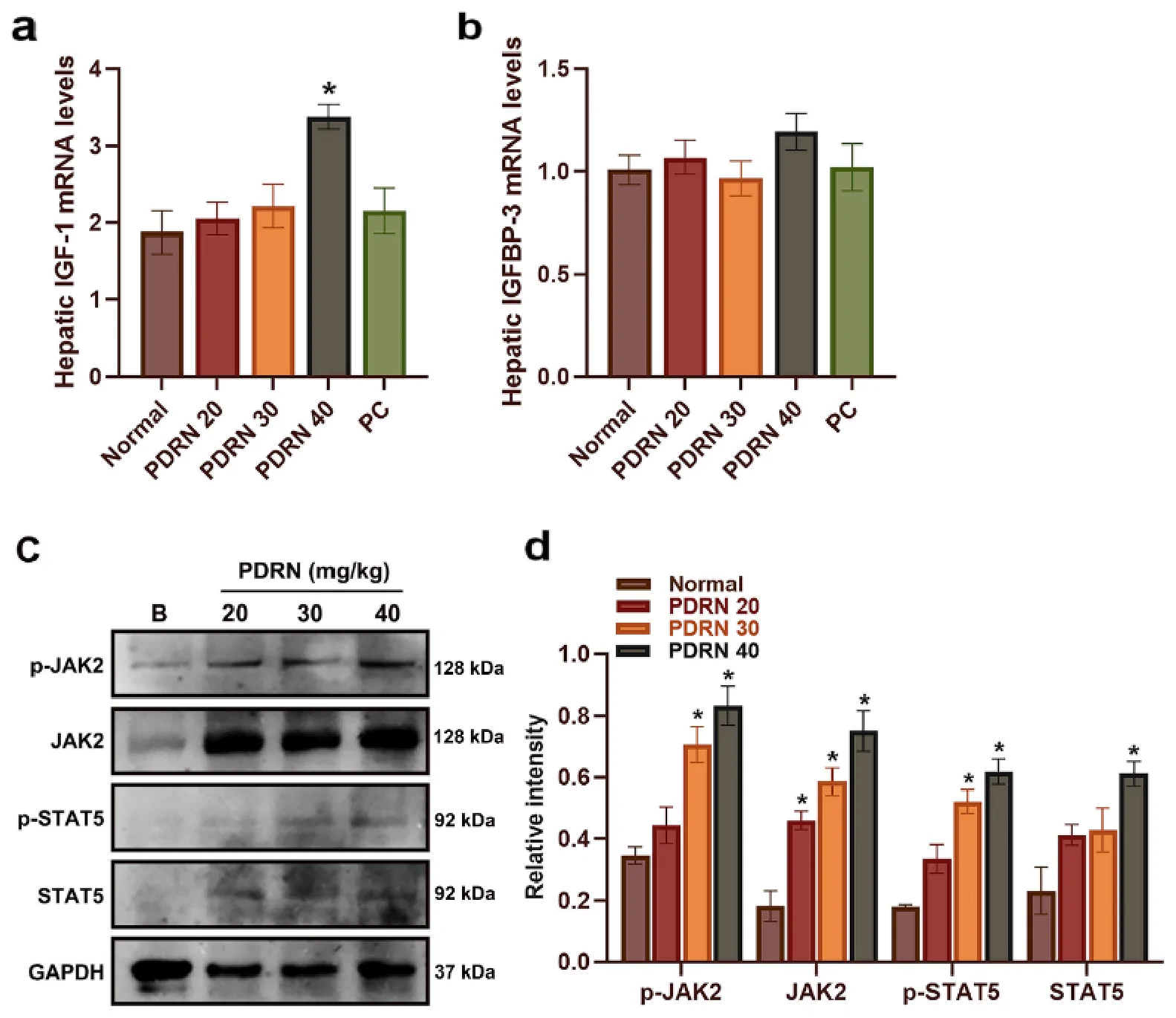
PDRN induces hepatic IGF-1 expression via JAK2-STAT5 signaling in vivo. Mouse liver tissues were homogenized, and (**a**) IGF-1 and (**b**) IGFBP-3 mRNA expression levels were analyzed by qRT-PCR. (**c**) The levels of p-JAK2, JAK2, p-STAT5, and STAT5 were determined by Western blotting, with representative images shown. (**d**) Protein expression levels were quantified by densitometry and normalized to GAPDH (p-JAK2/GAPDH, JAK2/GAPDH, p-STAT5/GAPDH, and STAT5/GAPDH). Values are presented as the mean ± SEM (*n* = 6 per group). * *p* < 0.05; significantly different from Normal. Normal, distilled water; PDRN 20, PDRN (20 mg/kg); PDRN 30, PDRN (30 mg/kg); PDRN 40, PDRN (40 mg/kg); PC, positive control (100 mg/kg).

Based on the in vitro findings showing that PDRN increased IGF-1 expression in association with A2AR-mediated JAK2-STAT5 activation, we next examined whether PDRN similarly affects hepatic IGF-1 expression and JAK2-STAT5 signaling in vivo. Because the liver is the primary source of circulating IGF-1 and a major site of IGF-1-binding protein-3 (IGFBP-3) production, we analyzed the expression of IGF-1, IGFBP-3, and JAK2-STAT5 signaling molecules in liver tissues. Hepatic IGF-1 mRNA expression was increased by PDRN, with a statistically significant upregulation observed specifically at 40 mg/kg compared to the normal group (Figure 2a; *p* < 0.05). Notably, PDRN at 40 mg/kg significantly increased IGF-1 expression without altering IGFBP-3 levels, whereas PC treatment did not significantly affect either marker (Figure 2a,b). Western blot analysis further showed that PDRN increased both total and p-JAK2 protein levels in liver tissues compared to the normal group (Figure 2c,d; *p* < 0.05). Consistently, protein levels of total STAT5 and p-STAT5 were also markedly elevated (Figure 2c,d; *p* < 0.05). Unlike the acute in vitro cellular response where total protein levels remained unchanged, chronic in vivo treatment significantly upregulated total JAK2 and STAT5 expression. To accurately reflect absolute expression changes without mathematical suppression caused by treatment-induced increases in total protein abundance, target bands in the in vivo analysis were normalized to glyceraldehyde 3-phosphate dehydrogenase (GAPDH) as an internal loading control.

### 2.3. PDRN Increases Systemic IGF-1 Levels and Promotes Longitudinal Growth and Growth Plate Expansion in Mice

**Figure 3:**
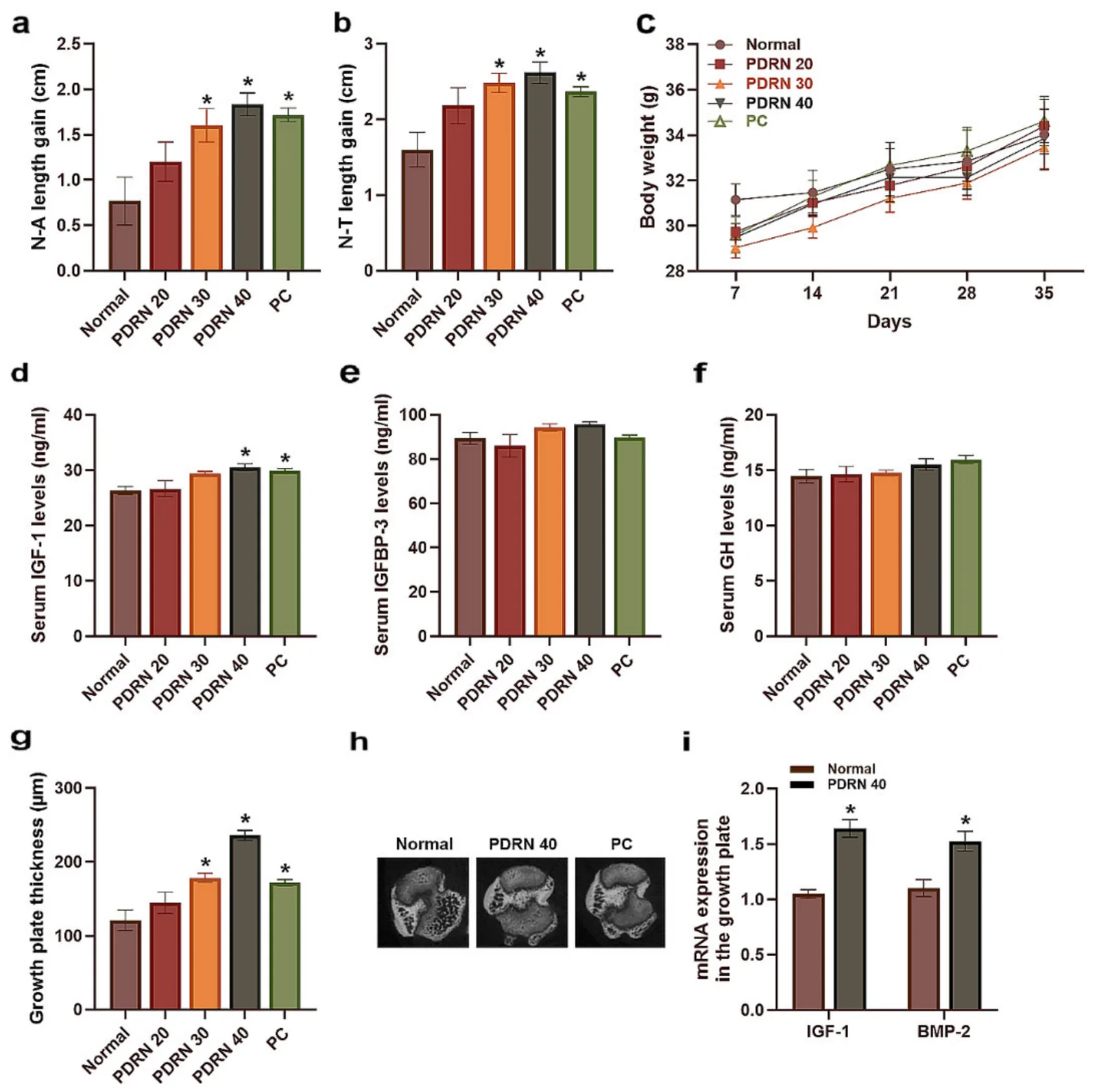
PDRN increases systemic IGF-1 levels, promotes linear growth, and activates the growth plate in mice. (**a**) N-A (nose-anus) length gain. (**b**) N-T (nose-tail) length gain. (**c**) Body weight. Serum levels of (**d**) IGF-1, (**e**) IGFBP-3, and (**f**) GH were determined by ELISA. (**g**) Growth plate thickness was quantified from 2D μCT slices. (**h**) Representative cross-sectional 2D μCT images showing the radiolucent growth plate area in the proximal tibia. (**i**) Relative mRNA expression levels of IGF-1 and BMP-2 in the growth plate were assessed by qRT-PCR. Values are presented as the mean ± SEM (*n* = 6 per group). * *p* < 0.05; significantly different from Normal. Normal, distilled water; PDRN 20, PDRN (20 mg/kg); PDRN 30, PDRN (30 mg/kg); PDRN 40, PDRN (40 mg/kg). PC, positive control (100 mg/kg).

We next investigated whether PDRN treatment promotes systemic growth in vivo. Body length and weight were monitored weekly for five weeks. As shown in Figure 3a,b, both nose-to-anus (N-A) and nose-to-tail (N-T) length gains were significantly higher in the PDRN 30 and 40 mg/kg groups than in the normal group (*p* < 0.05). While both PDRN 30 and 40 mg/kg significantly promoted N-A and N-T length gains compared to the normal group, there was no statistically significant difference between the PDRN 30 and 40 mg/kg groups. Additionally, these increases occurred without significant changes in body weight across all groups (Figure 3c). Similarly, the PC group exhibited a significant increase in body length without body weight changes (Figure 3a–c). The PDRN group showed no significant changes in serum parameters, including alanine transaminase (ALT), aspartate aminotransferase (AST), blood urea nitrogen (BUN), creatine kinase (CK), lactate dehydrogenase (LDH), and glucose (Supplementary Table S1).

Enzyme-linked immunosorbent assay (ELISA) measurements revealed that serum IGF-1 levels were significantly elevated in the PDRN-treated groups (Figure 3d; *p* < 0.05), whereas serum IGFBP-3 and GH levels remained unchanged (Figure 3e,f). Histological analysis of the proximal tibial growth plate showed that PDRN treatment significantly increased growth plate thickness and expanded the radiolucent band (Figure 3g,h; *p* < 0.05). Furthermore, mRNA levels of IGF-1 and bone morphogenetic protein-2 (BMP-2) in the growth plate were significantly higher in the PDRN group than in the normal group (Figure 3i; *p* < 0.05).

### 2.4. PDRN Improves Tibial and Femoral Bone Microarchitecture in Mice

**Figure 4:**
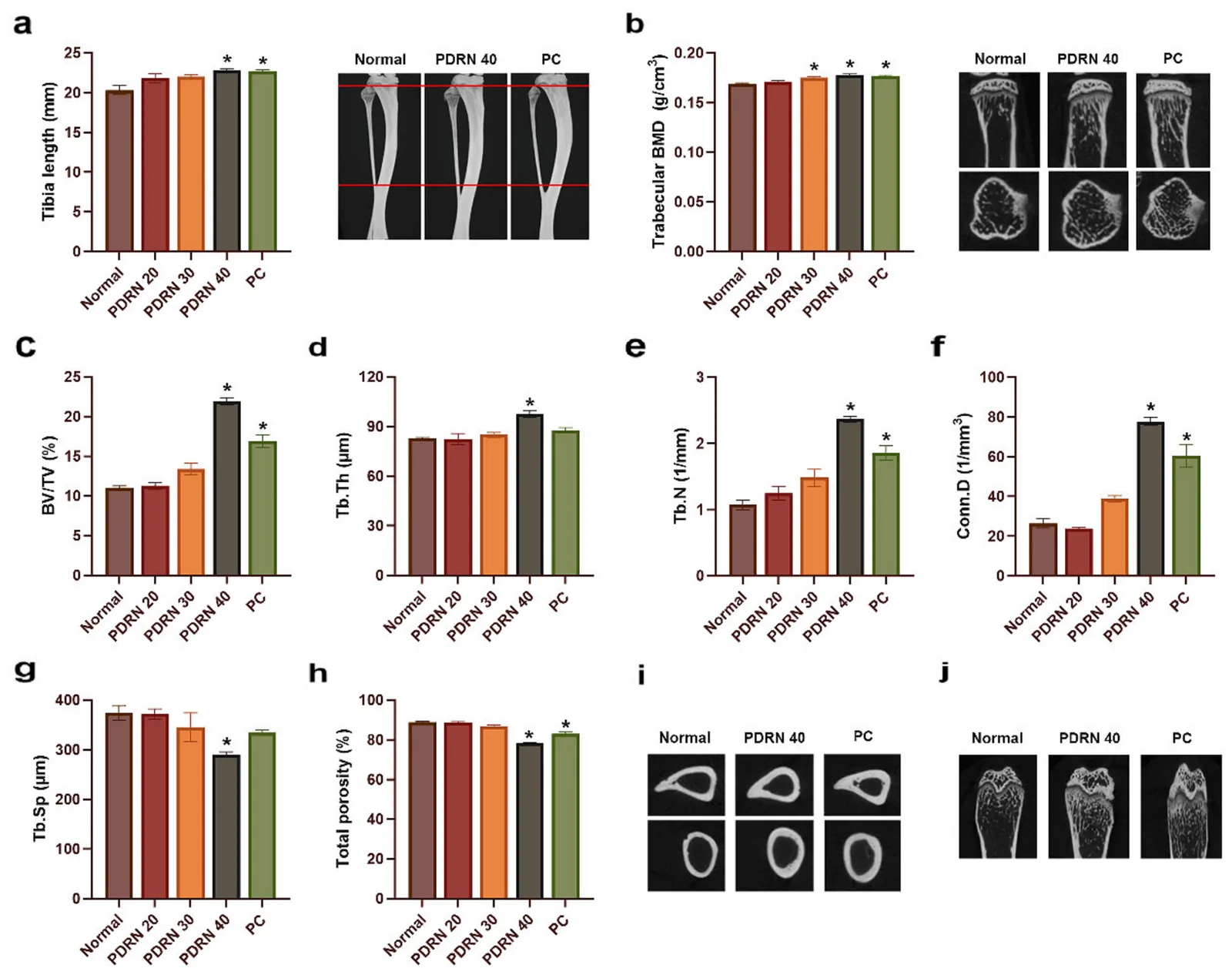
PDRN improves bone microarchitecture in mice. (**a**) Tibial length (left) and representative μCT images (right). (**b**) Trabecular BMD (left) and representative μCT images (right) of the tibia. Quantitative analysis of trabecular parameters: (**c**) BV/TV, (**d**) Tb.Th, (**e**) Tb.N, (**f**) Conn.D, (**g**) Tb.Sp, and (**h**) total porosity in the proximal tibia. (**i**) Tibial cortical BMD with representative μCT images. (**j**) Femoral BMD with representative μCT images. Values are presented as the mean ± SEM (*n* = 6 per group). * *p* < 0.05; significantly different from Normal. Normal, distilled water; PDRN 20, PDRN (20 mg/kg); PDRN 30, PDRN (30 mg/kg); PDRN 40, PDRN (40 mg/kg); PC, positive control (100 mg/kg).

To determine whether PDRN-associated growth promotion was accompanied by improvements in bone microarchitecture, quantitative microcomputed tomography μCT analysis was performed. PDRN treatment significantly increased tibial length and trabecular bone mineral density (BMD) (Figure 4a,b; *p* < 0.05). Key trabecular parameters, including bone volume fraction (BV/TV), trabecular thickness (Tb.Th), trabecular number (Tb.N), and connectivity density (Conn.D), were significantly higher in the PDRN groups compared to the normal group (Figure 4c–f; *p* < 0.05). Conversely, trabecular spacing (Tb.Sp) and total porosity were significantly reduced (Figure 4g,h; *p* < 0.05). While the PC group exhibited improvements in various indices, no significant differences were observed in Tb.Th and Tb.Sp. Furthermore, both tibial cortical BMD and femoral BMD in the high-dose PDRN group (40 mg/kg) were markedly higher than those of the normal group (Figure 4i,j).

### 2.5. PDRN Modulates Bone Remodeling-Related Responses by Suppressing Osteoclastogenic Markers and Enhancing Osteogenic Markers

**Figure 5:**
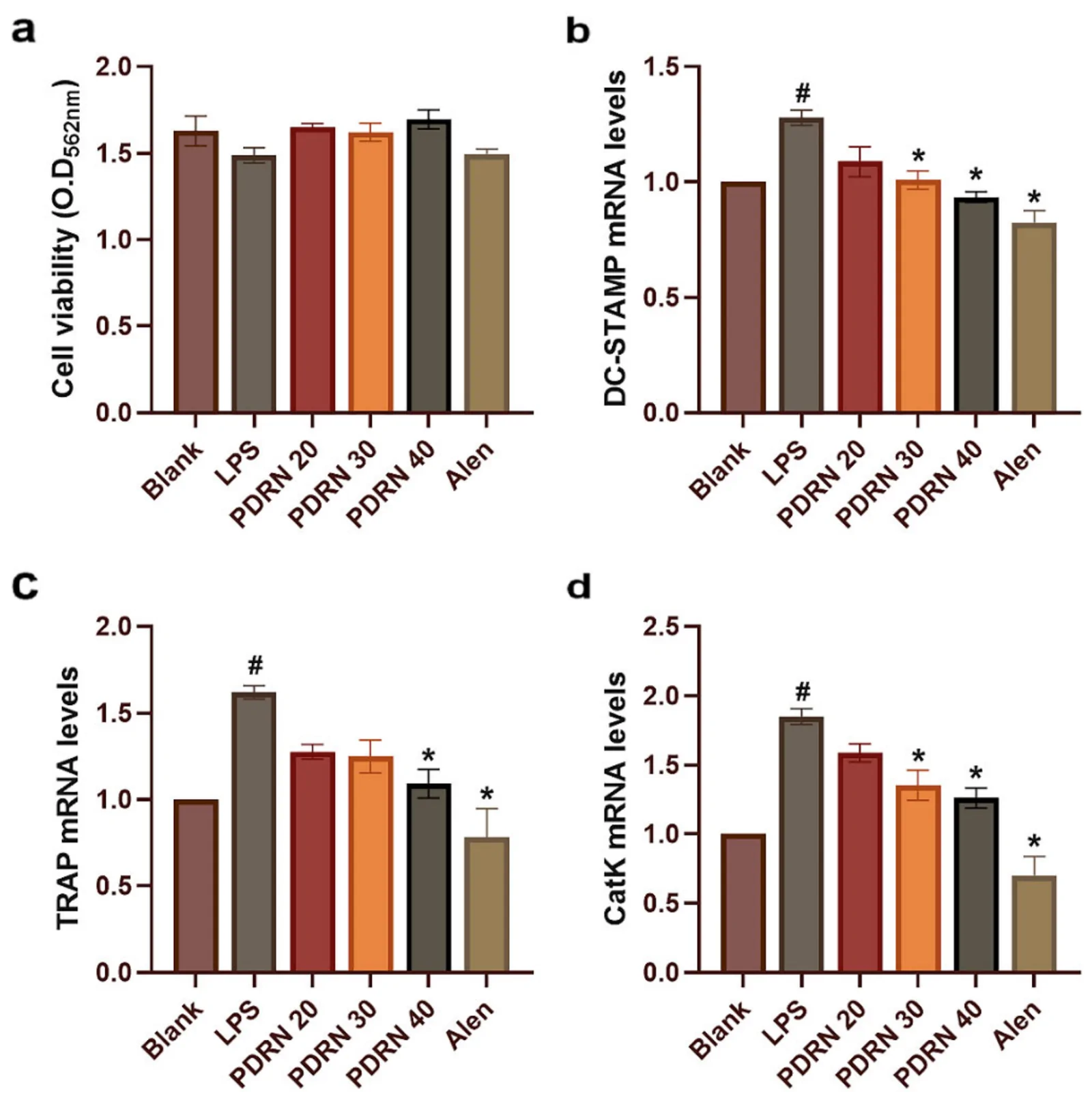
PDRN suppresses osteoclast-related gene expression in LPS-stimulated RAW 264.7 cells. RAW 264.7 cells (5 × 10^5^ cells/well) were pretreated with PDRN (20, 30, or 40 μg/mL) or Alendronate (100 μM) for 1 h before stimulation with LPS (100 ng/mL) for 48 h. (**a**) Cell viability was assessed using the MTT assay. After 24 h of LPS treatment, (**b**) DC-STAMP, (**c**) TRAP, and (**d**) CatK mRNA expression levels were assessed by qRT-PCR. Values are presented as the mean ± SEM. ^#^ *p* < 0.05, indicating a significant difference from the Blank. * *p* < 0.05, indicating a significant difference from the LPS stimulation. Blank, vehicle-treated cells; LPS, cells treated with LPS only; PDRN 20, LPS-treated cells pretreated with PDRN (20 μg/mL); PDRN 30, LPS-treated cells pretreated with PDRN (30 μg/mL); PDRN 40, LPS-treated cells pretreated with PDRN (40 μg/mL); Alen, LPS-treated cells pretreated with Alendronate (100 μM).

Finally, to explore cellular responses potentially contributing to the structural bone improvements observed in vivo, we evaluated the effects of PDRN on bone remodeling-related responses using RAW 264.7 osteoclast precursor cells and MC3T3-E1 pre-osteoblasts. In RAW 264.7 cells, PDRN did not significantly affect cell viability (Figure 5a). Lipopolysaccharide (LPS) markedly induced the mRNA expression of osteoclast-related markers, including dendritic cell-specific transmembrane protein (DC-STAMP), tartrate-resistant acid phosphatase (TRAP), and Cathepsin K (CatK), compared to the blank group (Figure 5b–d; *p* < 0.05). PDRN treatment significantly suppressed this LPS-induced upregulation in a dose-dependent manner (Figure 5b–d; *p* < 0.05).

**Figure 6:**
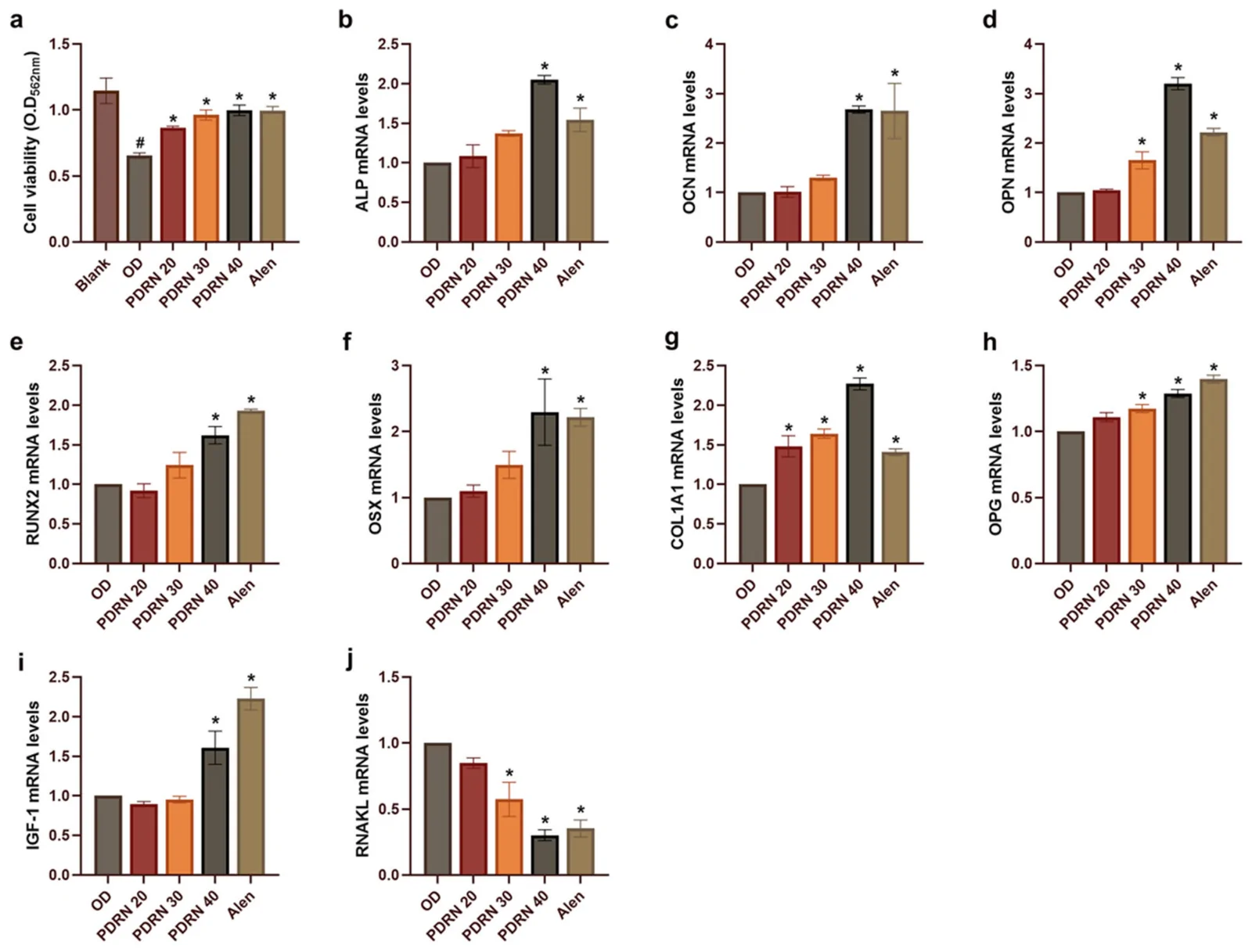
PDRN enhances osteogenic gene expression in MC3T3-E1 cells. MC3T3-E1 cells (1 × 10^4^ cells/well) were cultured in OD medium containing PDRN (20, 30, or 40 μg/mL) or Alendronate (10 μM) for 7 days. (**a**) Cell viability was assessed using the MTT assay. (**b**) ALP, (**c**) OCN, and (**d**) OPN mRNA expression levels were assessed by qRT-PCR. MC3T3-E1 cells (5 × 10^5^ cells/well) were cultured in OD medium containing PDRN (20, 30, or 40 μg/mL) and Alendronate (10 μM) for 3 days. (**e**) RUNX2, (**f**) OSX, (**g**) COL1A1, (**h**) OPG (**i**) IGF-1, and (**j**) RANKL mRNA expression levels were assessed by qRT-PCR. Values are presented as the mean ± SEM. ^#^ *p* < 0.05, indicating a significant difference from the Blank. * *p* < 0.05, indicating a significant difference from the OD medium. OD, OD medium; PDRN 20, OD medium plus PDRN (20 μg/mL); PDRN 30, OD medium plus PDRN (30 μg/mL); PDRN 40, OD medium plus PDRN (40 μg/mL); Alen, OD medium plus Alendronate (10 μM).

In MC3T3-E1 cells cultured under OD conditions, PDRN treatment significantly rescued cell viability compared to the OD group (Figure 6a; *p* < 0.05). Furthermore, PDRN significantly increased the expression of osteogenesis- and bone-remodeling-related genes, including alkaline phosphatase (ALP), osteocalcin (OCN), osteopontin (OPN), runt-related transcription factor 2 (RUNX2), osterix (OSX), collagen type I alpha 1 (COL1A1), osteoprotegerin (OPG), and IGF-1, while significantly suppressing receptor activator of nuclear factor-κB ligand (RANKL) expression (Figure 6b–j; *p* < 0.05). Together, these findings suggest that PDRN may contribute to bone accrual through modulation of osteoclast- and osteoblast-related gene expression.

## 3. Discussion

Bone growth and development are fundamental physiological processes that are regulated by complex interactions between hormonal, nutritional, and genetic factors [21]. During the growth phase, longitudinal bone growth occurs primarily through endochondral ossification within the cartilaginous growth plate [22]. Although the GH-IGF-1 axis has been established as a principal endocrine pathway for skeletal development, growing evidence indicates that linear growth may also involve localized or complementary signaling mechanisms alongside classical circulating GH action [6,11]. In the present study, oral PDRN promoted longitudinal bone growth in growing mice and was associated with increased hepatic and circulating IGF-1 levels, growth plate expansion, improved bone microarchitecture, and favorable changes in bone remodeling-related responses.

In the present study, PDRN promoted IGF-1 expression in association with A2AR signaling through the JAK2-STAT5 pathway. In HepG2 cells, PDRN treatment increased the phosphorylation of JAK2 and STAT5 without altering their total protein levels, accompanied by increased IGF-1 mRNA expression. These effects were significantly attenuated by the selective A2AR antagonist ZM241385, supporting the involvement of A2AR signaling in PDRN-induced JAK2-STAT5 activation and IGF-1 expression. However, these in vitro findings were obtained using HepG2 cells, a hepatocellular carcinoma cell line, and therefore may not fully represent IGF-1 regulation in normal hepatocytes. Accordingly, the observed A2AR-associated JAK2-STAT5/IGF-1 response should be interpreted within the context of the HepG2 cell model, and further studies using normal or primary hepatocytes are warranted to confirm this mechanism. Extending these findings using an in vivo model, chronic oral administration of PDRN for 5 weeks increased hepatic IGF-1 mRNA expression and the phosphorylation levels of JAK2 and STAT5 in mouse liver tissue. Unlike the acute in vitro response, in which total JAK2 and STAT5 protein levels were unchanged, chronic in vivo treatment increased both total and phosphorylated JAK2 and STAT5 levels. Because total protein expression was altered by chronic treatment, target protein levels were normalized to GAPDH to reflect absolute expression changes accurately without obscuring the net elevation in phosphorylated signaling components. Because these measurements were obtained at a single endpoint, the mechanism underlying the increase in total JAK2 and STAT5 remains unclear and requires further investigation. Since the liver serves as the predominant source of circulating IGF-1 [9,23], the increased hepatic IGF-1 expression observed following PDRN treatment may contribute to the elevation of circulating IGF-1 levels. The absence of changes in IGFBP-3 expression further suggests that PDRN-induced increases in IGF-1 were not accompanied by detectable changes in IGFBP-3 expression under the conditions examined. However, it should be noted that while the A2AR antagonist was utilized in vitro to clarify receptor involvement, no genetic or pharmacologic inhibition of A2AR, JAK2, or STAT5 was conducted in the mouse model. Consequently, our in vivo mechanistic findings remain correlative, and further studies utilizing specific inhibitors or knockout models in vivo are required to definitively prove the causal role of the A2AR-JAK2-STAT5 axis in PDRN-induced bone growth.

To translate these hepatic and systemic changes into growth-related outcomes, oral PDRN was administered for 5 weeks and was found to significantly increase both N-A and N-T length gains in mice without affecting overall body weight or serum biochemical parameters, including ALT, AST, BUN, CK, LDH, and glucose. These findings suggest that PDRN may promote longitudinal growth without inducing noticeable alterations in the measured serum biochemical parameters under the present experimental conditions. However, given the relatively limited sample size, further large-scale studies may be warranted to fully confirm these safety profiles. Importantly, serum IGF-1 levels were significantly elevated, whereas basal circulating GH and IGFBP-3 levels remained unchanged. These findings suggest that PDRN may enhance circulating IGF-1 availability without noticeably altering baseline systemic GH levels under the examined conditions. However, because serum GH was assessed at a single time point, potential alterations in GH pulse frequency, GHR expression, or downstream GHR signaling were not evaluated in this study. Furthermore, GH signaling was not directly inhibited or genetically disrupted. Therefore, unchanged basal serum GH levels alone are insufficient to definitively establish a GH-independent or GH-sparing mechanism, and further studies focusing on GH pulsatility and GHR responsiveness are required.

The present study provides evidence that orally administered PDRN can exert systemic growth-related effects in a growing mouse model. Although PDRN has been primarily investigated in the context of local tissue repair, the present findings extend its potential biological effects to systemic skeletal growth. However, because PDRN is a high-molecular-weight DNA polymer, the extent to which orally administered material is absorbed intact or converted into active metabolites (e.g., nucleosides or nucleotides) remains a central pharmacokinetic question underlying its proposed systemic mechanism. Without direct absorption, stability, or metabolite quantification data in the present study, the claim of direct hepatic A2AR activation by intact PDRN after oral gavage remains speculative. It is plausible that orally ingested PDRN undergoes enzymatic degradation in the gastrointestinal tract into lower-molecular-weight oligonucleotides, nucleosides (such as adenosine), or free bases, which are subsequently absorbed via intestinal nucleoside transporters and enter systemic circulation. Once distributed to tissues, these metabolites can replenish intracellular nucleotide pools via nucleotide-salvage pathways or activate membrane-bound adenosine receptors, including A2AR, to stimulate downstream signaling cascades. This limitation requires careful consideration when interpreting our in vivo findings and assessing the translational relevance of oral PDRN. Although systemic and hepatic responses were observed following oral administration, whether these effects stem from direct systemic exposure to intact PDRN or from its circulating active metabolic products remains to be clarified. Further studies are therefore critically needed to determine the absorption, biodistribution, and metabolism profile of PDRN following oral administration and to fully characterize its pharmacokinetic properties.

In addition to pharmacokinetic considerations, potential sex differences and translational caveats must be noted regarding pediatric applications. First, because only male mice were used to minimize prepubertal hormonal fluctuations, whether female animals exhibit identical sensitivity to oral PDRN remains to be established, particularly given the influence of sex steroids on growth plate dynamics and skeletal maturation. Second, translating these findings to pediatric clinical settings requires caution; unlike humans, mice lack true epiphyseal closure and exhibit continuous growth plate activity. Therefore, further studies incorporating female models, long-term safety evaluations, and precise dosage translation are necessary before considering human clinical application.

Locally within the growth plate, PDRN significantly increased total growth plate thickness, expanded the radiolucent cartilage region, and increased local mRNA expression of IGF-1 and BMP-2, both of which are involved in the regulation of chondrocyte proliferation, differentiation, and endochondral ossification [8,24]. Furthermore, quantitative μCT analysis demonstrated that PDRN treatment significantly improved BMD and microarchitectural parameters, including BV/TV, Tb.Th, Tb.N, and Conn.D, while reducing porosity in the tibia and femur. These findings indicate that the growth-promoting effects of PDRN were accompanied by improvements in bone structural properties. In addition, while total growth plate thickness was precisely quantified using high-resolution 2D μCT cross-sections, individual sub-zones (i.e., proliferative versus hypertrophic zones) were not distinctively separated due to the non-destructive nature of μCT imaging. Further histological studies with specific zonal marker staining will help clarify the precise cellular sub-zone affected by PDRN.

The improvements in bone architecture were further supported by cellular findings indicating that PDRN modulated bone remodeling-related responses. In RAW 264.7 cells, PDRN suppressed LPS-induced expression of osteoclast-related markers, including DC-STAMP, TRAP, and CatK. In MC3T3-E1 cells, PDRN increased the expression of osteogenesis- and bone-remodeling-related genes, including ALP, OCN, OPN, RUNX2, OSX, COL1A1, OPG, and IGF-1, while reducing RANKL expression. Together, these findings suggest that PDRN may contribute to bone accrual through the modulation of osteoclast- and osteoblast-related gene expression. These results further support recent research demonstrating that pharmacological attenuation of RANKL/RANK signaling and downstream NFATc1 activation plays a crucial role in inhibiting osteoclast maturation and mitigating structural bone damage [25]. However, because these cellular experiments primarily assessed molecular marker expression rather than direct osteoclast formation or mineralized matrix deposition, the functional effects of PDRN on osteoclastogenesis and osteoblast differentiation require further confirmation through functional assays, such as tartrate-resistant acid phosphatase staining and matrix mineralization assays.

To explore the potential for enhanced efficacy, we investigated whether the combined administration of low-dose PDRN with Ca or Mg could further influence growth-related outcomes. As these minerals are essential regulators of bone metabolism [26], the combination treatments increased IGF-1 mRNA expression in HepG2 cells and improved several bone-related parameters, including N-A and N-T length gains (Supplementary Figures S1 and S2). Additionally, PDRN, Ca, and Mg were not associated with significant changes in the measured biochemical safety parameters (Supplementary Table S1). These findings suggest that mineral supplementation may influence the growth-promoting effects of PDRN. However, the present findings do not establish a synergistic interaction, because formal interaction analyses and systematic dose–response comparisons were not performed. Further studies directly comparing PDRN alone, each mineral alone, and their combinations, together with appropriate synergy analyses, are necessary to determine whether these treatments exert additive, synergistic, or independent effects.

## 4. Materials and Methods

### 4.1. Preparation of PDRN

PDRN (purity > 80%) was provided by COSMAX NBT, INC. (Seongnam, Republic of Korea). Briefly, PDRN was isolated from salmon milt via extraction, filtration, concentration, and enzymatic treatment, and the resulting PDRN was confirmed to have a size distribution ranging from 150 to 400 bp (corresponding to approximately 97.5–260 kDa). The PDRN powder was dissolved in distilled water at 60 °C and filtered through a 0.22 μm surfactant-free cellulose acetate syringe filter (Sartorius, Göttingen, Germany).

### 4.2. Cell Culture

HepG2 (human hepatocellular carcinoma cell line), RAW 264.7 (murine pre-osteoclastic cell line), and MC3T3-E1 (murine pre-osteoblastic cell line) cells were purchased from the Korean Cell Line Bank (Seoul, Republic of Korea). HepG2 and RAW 264.7 cells were cultured in Dulbecco’s Modified Eagle’s Medium, and MC3T3-E1 cells were cultured in α-Minimum Essential Medium (Gibco BRL, Grand Island, NY, USA). All media were supplemented with 10% fetal bovine serum (FBS, Gibco BRL) and antibiotics (100 U/mL penicillin and 100 μg/mL streptomycin). Cells were maintained at 37 °C in a humidified incubator with 5% CO_2_ and at 95% humidity. RAW 264.7 cells were stimulated with LPS (100 ng/mL). Osteoblastic maturation of MC3T3-E1 cells was promoted using osteogenic differentiation (OD) medium containing 1 nM dexamethasone, 10 mM β-glycerophosphate, and 50 μg/mL ascorbic acid, with fresh OD medium replenished every 2–3 days during the differentiation protocol. Regular screening for mycoplasma contamination was performed using an e-Myco Mycoplasma PCR Detection Kit (iNtRON Biotechnology, Seongnam, Republic of Korea). To evaluate A2AR participation, HepG2 cells were pre-incubated with the selective antagonist ZM241385 (1 μM; Cayman Chemical, Ann Arbor, MI, USA) for 30 min prior to PDRN exposure.

### 4.3. MTT Assay

MTT assay was used to determine the viability of HepG2, RAW 264.7, and MC3T3-E1 cells. The plates were exposed to an MTT solution (0.5 mg/mL, Sigma-Aldrich, St. Louis, MO, USA) and incubated for 4 h. Subsequently, the insoluble formazan crystals were dissolved in dimethyl sulfoxide. The formazan content was quantified by measuring the absorbance at 562 nm with a microplate reader.

### 4.4. Quantitative Real-Time Polymerase Chain Reaction (qRT-PCR)

Total RNA was extracted from HepG2, RAW 264.7, and MC3T3-E1 cells, liver tissues, and growth plates using an easy-BLUE^TM^ RNA extraction kit (iNtRON Biotechnology, Sungnam, Gyeonggi-do, Republic of Korea). RNA concentration was measured using a NanoDrop spectrophotometry (Thermo Fisher Scientific Inc., Waltham, MA, USA), and cDNA was synthesized (Bioneer Corporation, Daejeon, Republic of Korea) using a reverse-transcription kit. qRT-PCR was performed using Power SYBR Green master mix on an ABI StepOne Real-Time PCR System (Applied Biosystems, Foster City, CA, USA). qRT-PCR was conducted to analyze human IGF-1, mouse IGF-1, IGFBP-3, BMP-2, DC-STAMP, TRAP, CatK, ALP, OCN, OPN, RUNX2, OSX, COL1A1, OPG, RANKL, and GAPDH. The data were calculated using the ΔΔCT method, employing GAPDH as the reference gene for normalization. Detailed primer sequences are shown in Supplementary Table S2.

### 4.5. Immunoblot Analysis

Western blot analysis was performed as previously described [27]. The liver tissue was homogenized in cold RIPA lysis buffer containing 1% protease inhibitor and 1% phosphatase inhibitor. A total of 150 μg of liver protein lysate was loaded per lane for Western blot analysis. The primary antibodies, including JAK2 (Santa Cruz Biotechnology, Dallas, TX, USA; sc-390539), phosphorylated (p)-JAK2 (Cell Signaling Technology, Danvers, MA, USA; #3771), STAT5 (Santa Cruz Biotechnology; sc-74442), p-STAT5 (Santa Cruz Biotechnology; sc-81524), and GAPDH (Santa Cruz Biotechnology; sc-32233), were diluted 1:500 in PBST. Protein bands were detected using the ImageQuant LAS 500 system (Cytiva, Marlborough, MA, USA), and band intensities were visualized with the ImageQuant TL software (version 10.1, Cytiva).

### 4.6. Animals and Ethical Statements

Four-week-old male ICR mice were obtained from DAMOOL SCIENCE (Daejeon, Republic of Korea). Only male mice were used in this study to minimize potential physiological variations associated with the female estrous cycle. The animals were housed in temperature- and humidity-controlled conditions (22 ± 2 °C, 55 ± 10% relative humidity) under a 12 h light/dark cycle, with ad libitum access to standard laboratory chow and water. All mice were acclimated for one week prior to experimental procedures. The mice were randomly assigned to five groups (N = 6 per group, total N = 30): Normal (distilled water), PDRN (20, 30, or 40 mg/kg), and PC (positive control, *Humulus japonicus* extract, 100 mg/kg). Treatments were administered via daily oral gavage for 35 consecutive days. The experimental unit was defined as an individual mouse. The sample size (N = 6 per group) was determined based on standard protocols in rodent growth models established in previous studies [1,7]. To validate the therapeutic efficacy of oral PDRN against an established reference, a patented non-hormonal composition containing standardized *Humulus japonicus* extract (rich in active flavonoids/polyphenols) was employed as a positive control (PC, 100 mg/kg). Recombinant human GH was not selected to avoid systemic hormonal interference and injection-related confounding factors in an oral administration model. The 100 mg/kg dose was selected based on prior evidence demonstrating its optimal efficacy in increasing IGF-1 expression and promoting longitudinal bone elongation [28]. While sharing the downstream effect of IGF-1 elevation with PDRN, this preparation serves as an orally active, non-hormonal reference standard. Animal health, body weight, and general behavior were monitored daily to ensure animal welfare. No animals exhibited signs of severe distress or unexpected adverse events, and no animals or data points were excluded from the analysis. To minimize bias, outcome measurements, including μCT analysis, histological evaluation, and serum biochemical assays/ELISAs, were performed by investigators blinded to group allocations. All animal experiments were conducted in compliance with the ARRIVE guidelines and performed in accordance with the NIH Guide for the Care and Use of Laboratory Animals. The animal study protocol was approved by the Institutional Animal Care and Use Committee (IACUC) of Jeonbuk National University (Approval No. NON2025-196-002, approved on 25 November 2025).

### 4.7. μCT

High-resolution μCT imaging was performed to evaluate osseous microarchitecture and longitudinal skeletal accretion, following established methodologies [8]. Briefly, to prevent tissue dehydration during image acquisition, isolated bone specimens were enveloped in parafilm and vertically positioned in a custom-built foam cartridge within an in vivo μCT system (SkyScan 1276; Bruker μCT, Kontich, Belgium). Scans were acquired at an X-ray source setting of 70 kV and 200 μA, utilizing a 0.5-mm aluminum filter. Projections were captured across a 180° rotation angle with 0.5° rotational increments, achieving an isotropic voxel resolution of 10.5 μm. Three-dimensional volumetric datasets were reconstructed using NRecon software 1.7.4.6 (Bruker μCT) and subsequently aligned along anatomical axes via DataViewer software 1.5.6.2 (Bruker μCT) to generate transaxial cross-sections for quantitative evaluation.

Regions of interest were delineated within the transaxial image stacks using CTAn software 1.16 (Bruker μCT). To analyze metaphyseal trabecular architecture at the proximal tibia, the trabecular compartment was manually traced slightly offset from the endocortical surface, beginning 0.4 mm distal to the growth plate metaphyseal border and spanning 50 consecutive slices. Segmentation of mineralized bone matrix from non-calcified tissue was achieved using a standardized global grayscale threshold (75–255). The measured trabecular microstructural indices comprised BV/TV, Conn.D, Tb.Th, Tb.Sp, Tb.N, and total porosity. Additionally, volumetric BMD of the trabecular tibia, cortical tibia, and femur was quantified. Linear tibial length was calculated as the straight-line distance spanning the proximal to distal epiphyses on reconstructed whole-bone projection scans. Growth plate height was evaluated on reconstructed 2D sagittal/coronal cross-sections of the proximal tibia using CTAn software, where the radiolucent physeal cartilage gap separating the distal epiphysis from the primary spongiosa was measured across multiple pre-defined reference coordinates.

### 4.8. ELISA

Recombinant protein and antibodies (IGF-1, 791-MG; IGFBP-3, 775-B3) were purchased from R&D Systems, Inc. (Minneapolis, MN, USA). The experimental assays were conducted in accordance with the protocol previously reported by Jeong et al. [29]. To measure IGF-1 and IGFBP-3 levels in serum, a modified sandwich ELISA method was used. Plates were coated overnight with capture antibodies (1 μg/mL; IGF-1, MAB791-SP; IGFBP-3, MAB775-SP). After coating, the plates were washed with phosphate-buffered saline (PBS) containing 0.05% Tween 20 (PBST) and blocked with 10% FBS in PBS for 2 h. In addition to washing, standard proteins or experimental samples were added and incubated for 2 h. Then, biotin-labeled secondary antibodies (1 μg/mL; IGF-1, BAF791; IGFBP-3, BAF775) were applied for 2 h, followed by avidin-peroxidase (diluted 1:400). The substrate solution was used, and absorbance was measured at 450 nm.

### 4.9. GH Assay

A mouse GH level in serum was analyzed according to the manufacturer’s specification (MyBioSource, Inc., San Diego, CA, USA, Cat. No. MBS160945). Briefly, serum samples and standards were added to pre-coated microplate wells and incubated to allow for GH binding. After washing to remove unbound substances, horseradish peroxidase-conjugated secondary antibodies were added. Following color development using a chromogenic substrate solution, the reaction was stopped, and absorbance was measured at 450 nm using a microplate reader.

### 4.10. Blood Biochemical Parameters

Biochemical parameters were measured using a DRI CHEM NX500 analyzer (Fujifilm, Tokyo, Japan). Levels of ALT, AST, BUN, CK, LDH, and glucose in the serum were determined.

### 4.11. Statistical Analysis

Statistical analyses were conducted using SPSS software (version 25.0; IBM Corp., Armonk, NY, USA). An a priori power analysis was conducted using G*Power software (version 3.1.9.7; Heinrich-Heine-University, Düsseldorf, Germany) to determine the sample size for in vivo experiments (*n* = 6 per group), which ensured an adequate statistical power (>80%) at a significance level of α = 0.05. Differences between two groups were evaluated by independent *t*-tests, and comparisons among multiple groups for endpoint parameters and net gains (N-A and N-T gains) were analyzed by one-way ANOVA followed by Dunnett’s post hoc test for comparisons against the control group. For longitudinally collected body weight data over the 35-day period, a two-way repeated-measures ANOVA was performed to evaluate time, treatment, and time × treatment interaction effects. All in vitro experiments were conducted in triplicate using independent trials. Data are expressed as the mean ± standard error of the mean (SEM) (*n* = 3 for in vitro independent experiments and *n* = 6 per group for in vivo studies), and exact *p*-values as well as effect sizes were evaluated and reported for key statistical comparisons (Supplementary Table S3). Statistical significance was set at *p* < 0.05 (indicated by * or #). Prism software was used for data visualization and graphical representations (version 8.0.1; GraphPad Software Inc., San Diego, CA, USA).

## 5. Conclusions

In conclusion, oral PDRN promoted longitudinal growth in growing mice, accompanied by increased hepatic and circulating IGF-1 levels, growth plate expansion, improved bone microarchitecture, and favorable changes in bone remodeling-related responses. In vitro assays demonstrated that PDRN activated JAK2-STAT5 signaling in association with A2AR signaling and increased IGF-1 expression in HepG2 cells, supporting an A2AR-associated hepatic mechanism that may contribute to its growth-promoting effects. However, these findings were obtained using HepG2 cells, and may not fully represent IGF-1 regulation in normal hepatocytes. The elevation of circulating IGF-1 without an increase in serum GH further suggests a potential GH-independent or GH-sparing component of PDRN-mediated growth promotion. Furthermore, because A2AR involvement was not directly tested in the in vivo model and whether PDRN directly affects growth plate chondrocytes through A2AR signaling remains to be determined, the exact contribution of systemic A2AR activation in vivo requires further investigation. Nonetheless, these findings suggest that oral PDRN may represent a potential approach for supporting longitudinal skeletal growth, and further studies are warranted to determine whether the observed hepatic mechanism is reproduced in normal hepatocytes, establish the contribution of A2AR signaling in vivo, clarify the direct effects of PDRN on growth plate chondrocytes, and evaluate its translational potential for skeletal growth, including sex-dependent responses and long-term safety in pediatric contexts.

## Data Availability

The original contributions presented in this study are included in the article. Further inquiries can be directed to the corresponding authors.

## Supplementary Materials

The following supporting information can be downloaded at https://www.mdpi.com/article/10.3390/molecules31193558/s1, Figure S1. Synergistic effects of PDRN supplemented with Ca or Mg on IGF-1 mRNA expression and body length. Figure S2. Synergistic effects of PDRN supplemented with Ca or Mg on the tibia and growth plate in mice. Table S1. Effects of PDRN on the blood biochemical parameters. Table S2. The sequence of primers used for qRT-PCR analysis. Table S3. Statistical analysis of pairwise comparisons among experimental groups. Unprocessed Western blot images.

## Abbreviations

A2AR	Adenosine A_2A_ receptor
ALT	Alanine transaminase
ALP	Alkaline phosphatase
AST	Aspartate aminotransferase
BUN	Blood urea nitrogen
BMD	Bone mineral density
BMP-2	Bone morphogenetic protein-2
BV/TV	Bone volume fraction
CatK	Cathepsin K
COL1A1	Collagen type I alpha 1
Conn.D	Connectivity density
CRE	Creatine kinase
DC-STAMP	Dendritic cell-specific transmembrane protein
FBS	Fetal bovine serum
GH	Growth hormone
GHR	Growth hormone receptor
IGF-1	Insulin-like growth factor-1
IGFBP-3	IGF-1 binding protein-3
JAK2	Janus kinase 2
LDH	Lactate dehydrogenase
LPS	Lipopolysaccharide
OD medium	Osteogenic differentiation medium
OCN	Osteocalcin
OPN	Osteopontin
OPG	Osteoprotegerin
OSX	Osterix
PDRN	Polydeoxyribonucleotide
RANKL	Receptor activator of nuclear factor-κB ligand
RUNX2	Runt-related transcription factor 2
STAT5	Signal transducer and activator of transcription 5
TRAP	Tartrate-resistant acid phosphatase
Tb.Sp	Trabecular spacing
Tb.N	Trabecular number
Tb.Th	Trabecular thickness
