# Supplementary material for: Polydeoxyribonucleotide Promotes Longitudinal Bone Growth Through the Hepatic JAK2-STAT5/IGF-1 Axis Involving A2A Receptor Signaling
Source: Molecules. 2026 Oct 7;31(19):3558. doi: 10.3390/molecules31193558 (PMC13666977; doi:10.3390/molecules31193558)

Polydeoxyribonucleotide Promotes Longitudinal Bone Growth Through the Hepatic JAK2-STAT5/IGF-1 Axis Involving A2A Receptor Signaling

Kyung-Min Jeong [1], Yu-Jin Choi [1], Seong-Been Yang [2], Minson Kweon [3], So-Young Rah [4,*] and Hyun-Ja Jeong[1,2,*]

1 Department of Bio-Convergence System, Hoseo University, 20 Hoseo-ro, 79 Beon-gil, Baebang-eup, Asan 31499, Chungcheongnam-do, Republic of Korea; juyk10@naver.com (K.-M.J.); dbwls9638@naver.com (Y.-J.C.)

2 Department of Food and Biotechnology, Graduate School, Hoseo University, Baebang-eup, Asan 31499, Chungcheongnam-do, Republic of Korea; sbin1597@naver.com

3 COSMAX NBT INC., Seongnam-si 13486, Gyeonggi-do, Republic of Korea; minson@cosmaxnbt.com

4 Department of Biochemistry and Molecular Biology, Jeonbuk National University Medical School, 20 Geonji-ro, Deokjin-gu, Jeonju-si 54907, Jeollabuk-do, Republic of Korea

* Correspondence: syrah76@jbnu.ac.kr (S.-Y.R.); hjjeong@hoseo.edu (H.-J.J.)

Supplementary methods

Animals

Male ICR mice (4-week-old) were obtained from the DAMOOL SCIENCE (Daejeon, Republic of Korea). The animals were acclimated and followed a 12-h light and 12-h dark cycle. Mice were orally administered PDRN (20 mg/kg, PDRN group) alone or in combination with Ca (PDRN 20 mg/kg + Ca 60 mg/kg, Ca group) or Mg (PDRN 14.5 mg/kg + Ca 43.6 mg/kg + Mg 21.8 mg/kg, Mg group) daily for 35 days (n = 6 per group). The dosages of these minerals were determined based on human equivalent doses (HED). Specifically, calcium and magnesium were administered at a 2:1 ratio (Ca:Mg) to reflect optimal physiological balance. All animal procedures and experiments were performed according to NIH guidelines for the care and use of laboratory animals. Protocols were approved by the Institutional Animal Care and Use Committee (IACUC) of Jeonbuk National University (JBU 2025-196-002).

Supplementary Figures

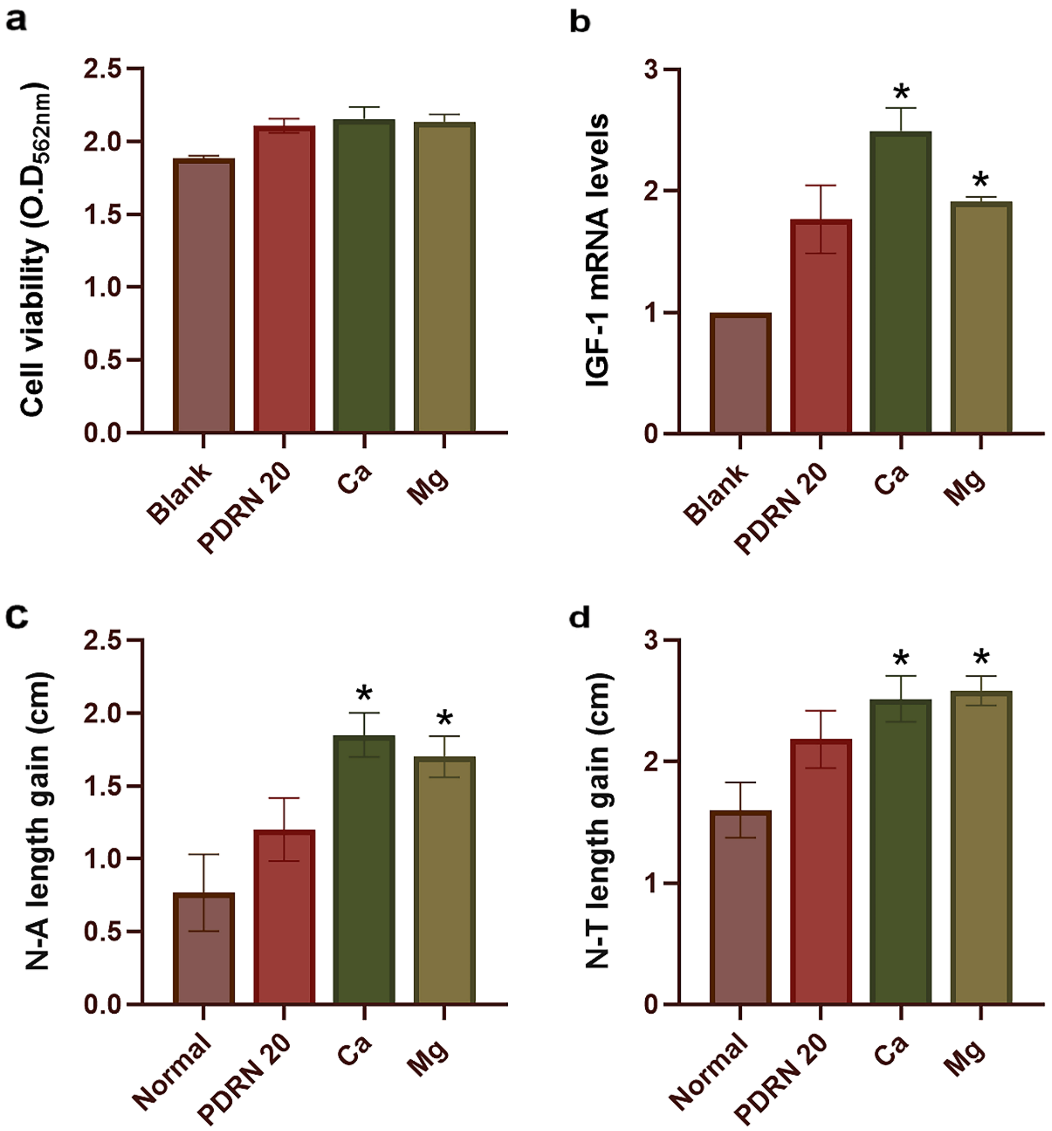

Supplementary Figure S1. Synergistic effects of PDRN supplemented with Ca or Mg on IGF-1 mRNA expression and body length. (**a**) HepG2 cells (1×10^4 cells/well) were treated with PDRN (20 μg/mL) alone or in combination with Ca (PDRN 20 μg/mL + 60 μg/mL) or Mg (PDRN 14.5 μg/mL + Ca 43.6 μg/mL + Mg 21.8 μg/mL) for 48 h. Cell viability was assessed using the MTT assay. (**b**) HepG2 cells (1×10^6 cells/well) were treated with PDRN (20 μg/mL) alone or in combination with Ca (PDRN 20 μg/mL + 60 μg/mL) or Mg (PDRN 14.5 μg/mL + Ca 43.6 μg/mL + Mg 21.8 μg/mL) for 24 h. IGF-1 mRNA expression was assessed by qRT-PCR. In animal model, (**c**) N-(**a**) length gain and (**d**) N-T length gain. Data are presented as the mean ± SEM. *$p < 0.05$; significantly different from Blank, vehicle-treated cells; Normal, distilled water

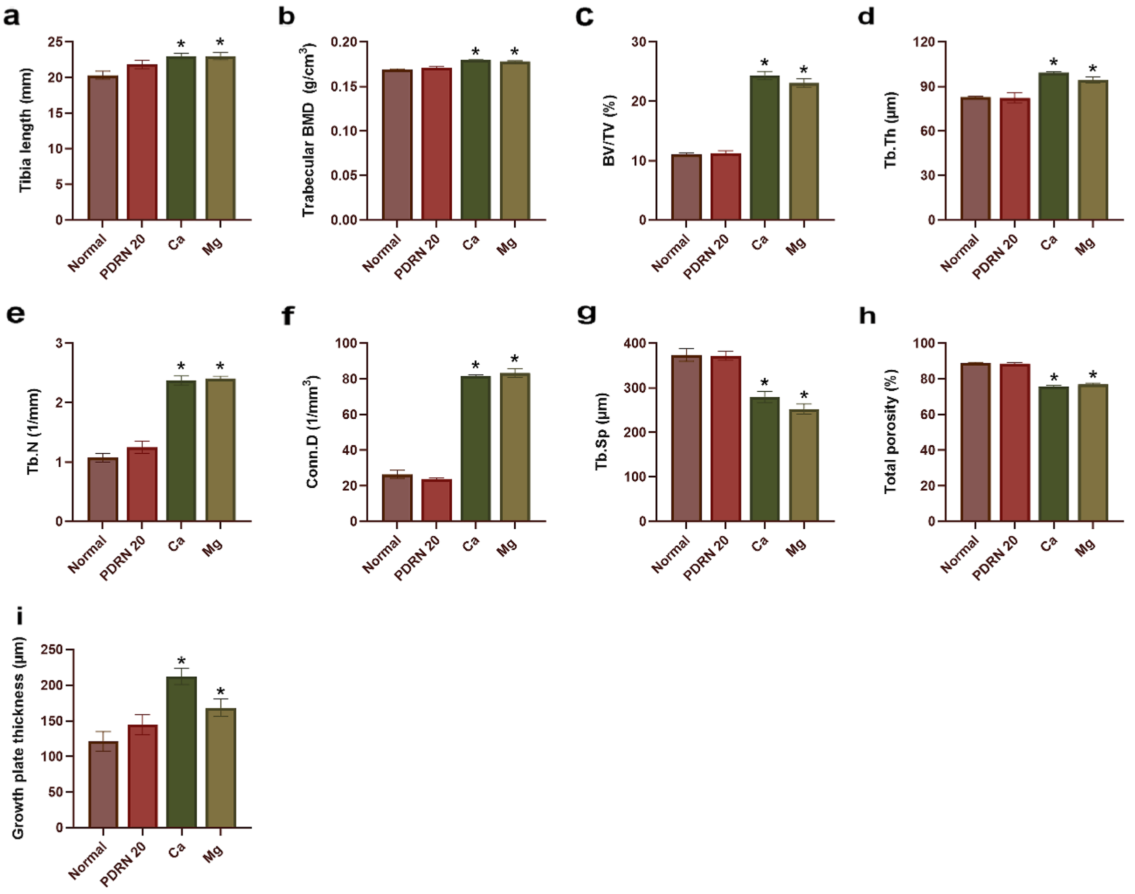

Supplementary Figure S2. Synergistic effects of PDRN supplemented with Ca or Mg on the tibia and growth plate in mice. (**a**) Tibia length, (**b**) Trabecular BMD, (**c**) BV/TV, (**d**) Tb.Th, (**e**) Tb.N, (**f**) Conn.D, (**g**) Tb.Sp, (**h**) total porosity, and (**i**) growth plate thickness. Data are presented as the mean ± SEM. *$p < 0.05$; significantly different from Normal. Normal, distilled water

Supplementary Tables

Supplementary Table S1. Effects of PDRN on the blood biochemical parameters.

	ALT (U/L)	AST (U/L)	BUN (mg/dL)	CK (mg/dL)	LDH (U/L)	Glucose (mg/dL)
Normal	75.50 ± 8.71	283.17 ± 44.23	19.65 ± 1.65	0.24 ± 0.027	1633.33 ± 274.06	733.33 ± 21.08
PDRN 20	63.00 ± 7.43	227.50 ± 38.88	18.60 ± 1.18	0.24 ± 0.038	1066.67 ± 170.62	700.00 ± 36.51
PDRN 30	61.50 ± 9.25	184.50 ± 52.40	19.60 ± 1.49	0.23 ± 0.025	1066.67 ± 91.89	733.33 ± 21.08
PDRN 40	59.50 ± 4.61	175.00 ± 34.19	18.10 ± 0.65	0.23± 0.044	983.33 ± 166.17	683.33 ± 16.67
Ca	67.50 ± 4.63	152.00 ± 22.11	18.60 ± 0.44	0.21 ± 0.014	1116.67 ± 157.94	716.67 ± 30.73
Mg	57.50 ± 3.14	208.00 ± 33.55	17.55 ± 1.20	0.19 ± 0.024	1400.00 ± 89.44	716.67 ± 16.67
PC	101.83 ± 24.30	283.17 ± 59.07	19.75 ± 2.31	0.18 ± 0.031	1900.00 ± 354.02	716.67 ± 16.67

Values are mean ± SEM. *$p < 0.05$ *vs.* Normal group. PC, positive control.

Supplementary Table S2. The sequence of primers used for qRT-PCR analysis

Genes		Sequences
Human IGF-1	Forward	TGCCCAAGACCCAGAAGT
	Reverse	CTCCTGTCCCCTCCTTCTGTT
Mouse IGF-1	Forward	CCCGGACCAGAGACCCTTTG
	Reverse	CCTGTGGGCTTGTTGAAGTAAAA
Mouse IGFBP-3	Forward	CCAGAACTTCTCCTCCGAGTCTAAG
	Reverse	CTCAGCACATTGAGGAACTTCAGAT
Mouse BMP-2	Forward	AAGCGTCAAGCCAAACACAAAC
	Reverse	GCCACGATCCAGTCATTCCAC
Mouse DC-STAMP	Forward	GGCCAACTCAAGAAGAAAAC
	Reverse	TCCTCCATGAACAAACAGTTCCAA
Mouse TRAP	Forward	ACACAGTGATGCTGTGTGGCAACTC
	Reverse	CCAGAGGCTTCCACATATATGATGG
Mouse CatK	Forward	GGCCAACTCAAGAAGAAAAC
	Reverse	GTGCTTGCTTCCCTTCTGG
Mouse ALP	Forward	TCGGGACTGGTACTCGGATAA
	Reverse	GTTCAGTGCGGTTCCAGACATAG
Mouse OCN	Forward	CTGACCTCACAGATCCCAAGC
	Reverse	TGGTCTGATAGCTCGTCACAAG
Mouse OPN	Forward	CCAACGGCCGAGGTGATA
	Reverse	CAGGCTGGCTTTGGAACTTG
Mouse RUNX2	Forward	AGACTGCAAGAAGGCTCTGG
	Reverse	TTCCTGCATGGACTGTGGTT
Mouse OSX	Forward	GTCCTCTCTGCTTGAGGAA
	Reverse	CTTGAGAAGGGAGCTGGGT
Mouse COL1A1	Forward	GCCTCCCAGAACATCACCTA
	Reverse	GCAGGGACTTCTTGAGGTTG
Mouse OPG	Forward	AAAGCACCCTGTAGAAAACA
	Reverse	CCGTTTTATCCTCTCTACACTC

Mouse RANKL	Forward	CCATCGGGTTCCCATAAAGTCA
	Reverse	CAGTTTTTCGTGCTCCCTCCTT
Human GAPDH	Forward	ACGCATTTGGTCGTATTGGG
	Reverse	TGATTTTGGAGGGATCTCGC
Mouse GAPDH	Forward	CATGTTCCAGTATGACTCCACTC
	Reverse	GGCCTCACCCCATTTGATGT

Supplementary Table S3. Statistical analysis of pairwise comparisons among experimental groups

		Groups	p value	Cohen's d (effect size)	
FIG1	b	Blank vs. PDRN 20 ug/ml	0.0163	2.71	
		Blank vs. PDRN 30 ug/ml	0.0002	5.08	
		Blank vs. PDRN 40 ug/ml	<0.0001	6.95	
		Blank vs. Positive Control	<0.0001	7.05	
	d	p-JAK2-JAK2: Blank vs. PDRN 40 ug/ml	0.0006	8.16	t-test
		p-JAK2-JAK2: PDRN 40 ug/ml vs. PDRN+ZM	0.0011	7.07	t-test
		p-STAT5/STAT5: Blank vs. PDRN 40 ug/ml	0.0003	9.53	t-test
		p-STAT5/STAT5: PDRN 40 ug/ml vs. PDRN+ZM	0.0002	10.87	t-test
	e	Blank vs. PDRN 40 ug/ml	0.0003	7.81	t-test
		PDRN 40 ug/ml vs. PDRN+ZM	0.0036	4.06	t-test
FIG2	a	Normal vs. PDRN 20mg/kg	0.9412	0.28	
		Normal vs. PDRN 30mg/kg	0.5986	0.53	
		Normal vs. PDRN 40mg/kg	P < 0.0001	2.31	
		Normal vs. Positive Control	0.7483	0.37	
	d	p-JAK2 / GAPDH: Normal vs. PDRN 20mg/kg	0.3807	1.31	
		p-JAK2 / GAPDH: Normal vs. PDRN 30mg/kg	0.0028	4.51	
		p-JAK2 / GAPDH: Normal vs. PDRN 40mg/kg	0.0004	5.79	
		JAK2 / GAPDH: Normal vs. PDRN 20mg/kg	0.0116	3.87	
		JAK2 / GAPDH: Normal vs. PDRN 30mg/kg	0.002	7.92	
		JAK2 / GAPDH: Normal vs. PDRN 40mg/kg	0.0004	5.57	
		p-STAT5 / GAPDH: Normal vs. PDRN 20mg/kg	0.0505	2.63	
		p-STAT5 / GAPDH: Normal vs. PDRN 30mg/kg	0.0004	7.18	
		p-STAT5 / GAPDH: Normal vs. PDRN 40mg/kg	P < 0.0001	8.51	
		STAT5 / GAPDH: Normal vs. PDRN 20mg/kg	0.1345	1.77	
		STAT5 / GAPDH: Normal vs. PDRN 30mg/kg	0.1706	1.55	
		STAT5 / GAPDH: Normal vs. PDRN 40mg/kg	0.0087	3.62	
FIG3	a	Normal vs. PDRN 20mg/kg	0.4855	0.99	
		Normal vs. PDRN 30mg/kg	0.01	2.05	
		Normal vs. PDRN 40mg/kg	0.0003	2.51	
		Normal vs. Positive Control	0.0018	2.57	

	b	Normal vs. PDRN 20mg/kg	0.1251	1.09	
		Normal vs. PDRN 30mg/kg	0.0121	1.81	
		Normal vs. PDRN 40mg/kg	0.0039	2.09	
		Normal vs. Positive Control	0.0322	1.86	
	d	Normal vs. PDRN 20mg/kg	0.9999	0.17	
		Normal vs. PDRN 30mg/kg	0.0894	1.84	
		Normal vs. PDRN 40mg/kg	0.0049	2.45	
		Normal vs. Positive Control	0.0215	2.05	
	g	Normal vs. PDRN 20mg/kg	0.7719	0.65	
		Normal vs. PDRN 30mg/kg	0.0116	2.05	
		Normal vs. PDRN 40mg/kg	P < 0.0001	4.13	
		Normal vs. Positive Control	0.0333	2.02	
	i	IGF-1; Normal vs. PDRN 40mg/kg	0.0025	5.47	t-test
		BMP2: Normal vs. PDRN 40mg/kg	0.0225	2.95	t-test
FIG4	a	Normal vs. PDRN 20mg/kg	0.2831	1.09	
		Normal vs. PDRN 30mg/kg	0.1743	1.63	
		Normal vs. PDRN 40mg/kg	0.0051	2.44	
		Normal vs. Positive Control	0.0118	2.27	
	b	Normal vs. PDRN 20mg/kg	0.6498	1.25	
		Normal vs. PDRN 30mg/kg	0.0022	2.87	
		Normal vs. PDRN 40mg/kg	P < 0.0001	4.31	
		Normal vs. Positive Control	0.0001	3.83	
	c	Normal vs. PDRN 20mg/kg	0.9998	0.31	
		Normal vs. PDRN 30mg/kg	0.1715	1.77	
		Normal vs. PDRN 40mg/kg	P < 0.0001	10.96	
		Normal vs. Positive Control	P < 0.0001	5.9	
	d	Normal vs. PDRN 20mg/kg	0.9356	0.07	
		Normal vs. PDRN 30mg/kg	0.9413	0.52	
		Normal vs. PDRN 40mg/kg	P < 0.0001	3.65	
		Normal vs. Positive Control	0.3418	1.19	

	e	Normal vs. PDRN 20mg/kg	0.9356	0.81	
		Normal vs. PDRN 30mg/kg	0.1586	1.58	
		Normal vs. PDRN 40mg/kg	P < 0.0001	6.42	
		Normal vs. Positive Control	0.0001	3.9	
	f	Normal vs. PDRN 20mg/kg	0.9997	-0.603	
		Normal vs. PDRN 30mg/kg	0.2806	2.591	
		Normal vs. PDRN 40mg/kg	P < 0.0001	9.94	
		Normal vs. Positive Control	0.0001	6.59	
	g	Normal vs. PDRN 20mg/kg	0.9999	-0.07	
		Normal vs. PDRN 30mg/kg	0.8568	-0.51	
		Normal vs. PDRN 40mg/kg	0.0054	-3.19	
		Normal vs. Positive Control	0.5392	-1.52	
	h	Normal vs. PDRN 20mg/kg	0.9998	-0.31	
		Normal vs. PDRN 30mg/kg	0.1715	-1.77	
		Normal vs. PDRN 40mg/kg	P < 0.0001	-10.96	
		Normal vs. Positive Control	P < 0.0001	-5.9	
FIG5	b	Blank vs. LPS	0.0011	6.87	t-test
		LPS vs. PDRN 20 ug/ml	0.2312	1.9	
		LPS vs. PDRN 30 ug/ml	0.05	4.07	
		LPS vs. PDRN 40 ug/ml	0.0145	7.39	
		LPS vs. Allendronate	0.002	5.82	
	c	Blank vs. LPS	0.0001	12.13	t-test
		LPS vs. PDRN 20 ug/ml	0.2393	3.69	
		LPS vs. PDRN 30 ug/ml	0.1873	2.45	
		LPS vs. PDRN 40 ug/ml	0.0401	4.14	
		LPS vs. Allendronate	0.0022	4.09	
	d	Blank vs. LPS	0.0001	13.19	t-test
		LPS vs. PDRN 20 ug/ml	0.4908	2.12	
		LPS vs. PDRN 30 ug/ml	0.049	3.31	
		LPS vs. PDRN 40 ug/ml	0.0203	4.88	
		LPS vs. Allendronate	0.0002	6.01	

FIG6	a	Blank vs. OD	0.0027	3.47	t-test
		OD vs. PDRN 20 ug/ml	0.0015	6.33	
		OD vs. PDRN 30 ug/ml	0.000026	5.04	
		OD vs. PDRN 40 ug/ml	0.000007	5.51	
		OD vs. Allendronate	0.000008	6.39	
	b	OD vs. PDRN 20 ug/ml	0.9718	0.47	
		OD vs. PDRN 30 ug/ml	0.1209	8.47	
		OD vs. PDRN 40 ug/ml	0.0001	15.98	
		OD vs. Allendronate	0.0177	3	
	c	OD vs. PDRN 20 ug/ml	0.9999	0.1	
		OD vs. PDRN 30 ug/ml	0.9223	4.48	
		OD vs. PDRN 40 ug/ml	0.0001	19.33	
		OD vs. Allendronate	0.0002	2.4	
	d	OD vs. PDRN 20 ug/ml	0.9966	1.88	
		OD vs. PDRN 30 ug/ml	0.00714	3.09	
		OD vs. PDRN 40 ug/ml	P < 0.0001	14.67	
		OD vs. Allendronate	0.0001	13.09	
	e	OD vs. PDRN 20 ug/ml	0.8521	-0.74	
		OD vs. PDRN 30 ug/ml	0.432	1.63	
		OD vs. PDRN 40 ug/ml	0.007	4.64	
		OD vs. Allendronate	0.0003	32.69	
	f	OD vs. PDRN 20 ug/ml	0.9932	0.69	
		OD vs. PDRN 30 ug/ml	0.612	1.34	
		OD vs. PDRN 40 ug/ml	0.0308	2.1	
		OD vs. Allendronate	0.042	7.34	
	g	OD vs. PDRN 20 ug/ml	0.00802	2.96	
		OD vs. PDRN 30 ug/ml	0.00101	8.78	
		OD vs. PDRN 40 ug/ml	0.00001	13.62	
		OD vs. Allendronate	0.02317	7.9	
	h	OD vs. PDRN 20 ug/ml	0.11	2.62	
		OD vs. PDRN 30 ug/ml	0.0088	4.67	
		OD vs. PDRN 40 ug/ml	0.0002	7.81	
		OD vs. Allendronate	P < 0.0001	10.91	

	i	OD vs. PDRN 20 ug/ml	0.92	-1.54	
		OD vs. PDRN 30 ug/ml	0.9921	-0.87	
		OD vs. PDRN 40 ug/ml	0.0259	2.37	
		OD vs. Allendronate	0.0002	7.12	
	j	OD vs. PDRN 20 ug/ml	0.55918	-3.15	
		OD vs. PDRN 30 ug/ml	0.00981	-2.7	
		OD vs. PDRN 40 ug/ml	0.00024	-14	
		OD vs. Allendronate	0.00045	-8.2	

Unprocessed Western blot images

Fig. 1c

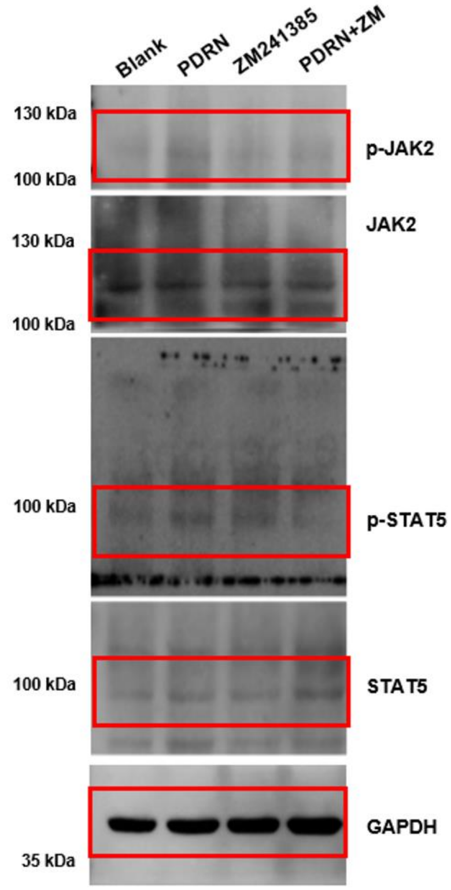

Fig. 2c

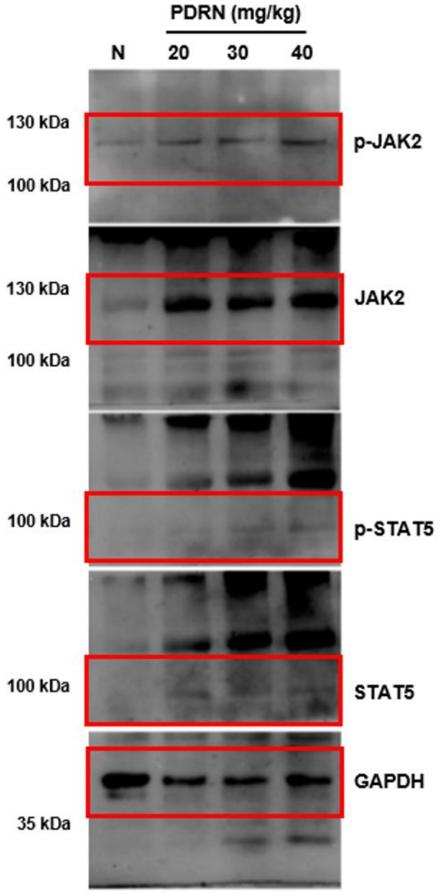

Supplement: Supplementary file 1 [file molecules-31-03558-s001.zip › molecules-4589382-supplementary.pdf]
